# HELM-GPT: *de novo* macrocyclic peptide design using generative pre-trained transformer

**DOI:** 10.1093/bioinformatics/btae364

**Published:** 2024-06-12

**Authors:** Xiaopeng Xu, Chencheng Xu, Wenjia He, Lesong Wei, Haoyang Li, Juexiao Zhou, Ruochi Zhang, Yu Wang, Yuanpeng Xiong, Xin Gao

**Affiliations:** Computer Science Program, Computer, Electrical and Mathematical Science and Engineering (CEMSE), King Abdullah University of Science and Technology (KAUST), Thuwal 23955-6900, Makkah, Kingdom of Saudi Arabia; Computational Bioscience Research Center, King Abdullah University of Science and Technology (KAUST), Thuwal 23955-6900, Makkah, Kingdom of Saudi Arabia; Computer Science Program, Computer, Electrical and Mathematical Science and Engineering (CEMSE), King Abdullah University of Science and Technology (KAUST), Thuwal 23955-6900, Makkah, Kingdom of Saudi Arabia; Computational Bioscience Research Center, King Abdullah University of Science and Technology (KAUST), Thuwal 23955-6900, Makkah, Kingdom of Saudi Arabia; Computer Science Program, Computer, Electrical and Mathematical Science and Engineering (CEMSE), King Abdullah University of Science and Technology (KAUST), Thuwal 23955-6900, Makkah, Kingdom of Saudi Arabia; Computational Bioscience Research Center, King Abdullah University of Science and Technology (KAUST), Thuwal 23955-6900, Makkah, Kingdom of Saudi Arabia; Computer Science Program, Computer, Electrical and Mathematical Science and Engineering (CEMSE), King Abdullah University of Science and Technology (KAUST), Thuwal 23955-6900, Makkah, Kingdom of Saudi Arabia; Computational Bioscience Research Center, King Abdullah University of Science and Technology (KAUST), Thuwal 23955-6900, Makkah, Kingdom of Saudi Arabia; Computer Science Program, Computer, Electrical and Mathematical Science and Engineering (CEMSE), King Abdullah University of Science and Technology (KAUST), Thuwal 23955-6900, Makkah, Kingdom of Saudi Arabia; Computational Bioscience Research Center, King Abdullah University of Science and Technology (KAUST), Thuwal 23955-6900, Makkah, Kingdom of Saudi Arabia; Computer Science Program, Computer, Electrical and Mathematical Science and Engineering (CEMSE), King Abdullah University of Science and Technology (KAUST), Thuwal 23955-6900, Makkah, Kingdom of Saudi Arabia; Computational Bioscience Research Center, King Abdullah University of Science and Technology (KAUST), Thuwal 23955-6900, Makkah, Kingdom of Saudi Arabia; Syneron Technology, Guangzhou 510000, China; Syneron Technology, Guangzhou 510000, China; Syneron Technology, Guangzhou 510000, China; Computer Science Program, Computer, Electrical and Mathematical Science and Engineering (CEMSE), King Abdullah University of Science and Technology (KAUST), Thuwal 23955-6900, Makkah, Kingdom of Saudi Arabia; Computational Bioscience Research Center, King Abdullah University of Science and Technology (KAUST), Thuwal 23955-6900, Makkah, Kingdom of Saudi Arabia

## Abstract

**Motivation:**

Macrocyclic peptides hold great promise as therapeutics targeting intracellular proteins. This stems from their remarkable ability to bind flat protein surfaces with high affinity and specificity while potentially traversing the cell membrane. Research has already explored their use in developing inhibitors for intracellular proteins, such as KRAS, a well-known driver in various cancers. However, computational approaches for *de novo* macrocyclic peptide design remain largely unexplored.

**Results:**

Here, we introduce HELM-GPT, a novel method that combines the strength of the hierarchical editing language for macromolecules (HELM) representation and generative pre-trained transformer (GPT) for *de novo* macrocyclic peptide design. Through reinforcement learning (RL), our experiments demonstrate that HELM-GPT has the ability to generate valid macrocyclic peptides and optimize their properties. Furthermore, we introduce a contrastive preference loss during the RL process, further enhanced the optimization performance. Finally, to co-optimize peptide permeability and KRAS binding affinity, we propose a step-by-step optimization strategy, demonstrating its effectiveness in generating molecules fulfilling both criteria. In conclusion, the HELM-GPT method can be used to identify novel macrocyclic peptides to target intracellular proteins.

**Availability and implementation:**

The code and data of HELM-GPT are freely available on GitHub (https://github.com/charlesxu90/helm-gpt).

## 1 Introduction

Current drugs are estimated to effectively target only 20% of all disease-relevant human proteins, with the majority of the remaining proteins primarily engaged in intracellular protein–protein interactions (Buyanova and Pei 2022). These proteins are conventionally considered “undruggable,” because they do not contain well-defined binding pockets for small molecules and are inaccessible to biologics, such as antibodies, due to the obstacle of the cell membrane (Buyanova and Pei 2022). KRAS is an example, which is an important target in cancer. It was initially considered undruggable due to its lack of suitable binding pockets and its intracellular location (Yang *et al.* 2022). A promising new class of therapeutics, macrocyclic peptides, are changing the game. Macrocyclic peptides are capable of binding to flat protein surface with high affinity and specificity and have the potential to pass through the cell membrane by passive diffusion (Buyanova and Pei 2022). They have emerged as an alternative kind of therapeutics for targeting such proteins in recent years (Buyanova and Pei 2022).

Structure-based methods were developed to design macrocyclic peptides with target binding affinity (Mulligan *et al.* 2021) and cell membrane permeability (Bhardwaj *et al.* 2022) alone, but not together. While generative models have revolutionized small molecule design (Meyers *et al.* 2021, Xu *et al.* 2024) and even shown promise in protein design (Strokach and Kim 2022, Xu *et al.* 2023), their application to macrocyclic peptides remains unexplored. Macrocyclic peptides contain non-natural amino acids and chemical bonds to cyclize linear peptides, defying full representation by mere amino acid sequences. Existing small molecule design methods often rely on the simplified molecular input line entry system (SMILES) or molecular graphs (Meyers *et al.* 2021). Though technically these methods can be applied to design macrocyclic peptides, they primarily thrive in the realm of smaller, simpler molecules, neglecting the specific challenges of peptide synthesis (Mulligan 2020). Macrocyclic peptides are usually synthesized through the one-by-one incorporation of natural or non-natural amino acids, and are followed by reactions to cyclize peptides (Qian *et al.* 2015). Generative models exploring SMILES or molecular graph space often generate an abundance of unsynthesizable molecules, hindering their usefulness for peptide design. Exploring such vast molecular spaces risks generating an overabundance of unsynthesizable peptides, ultimately resulting in fruitless wet-lab experiments.

**Figure 1.**
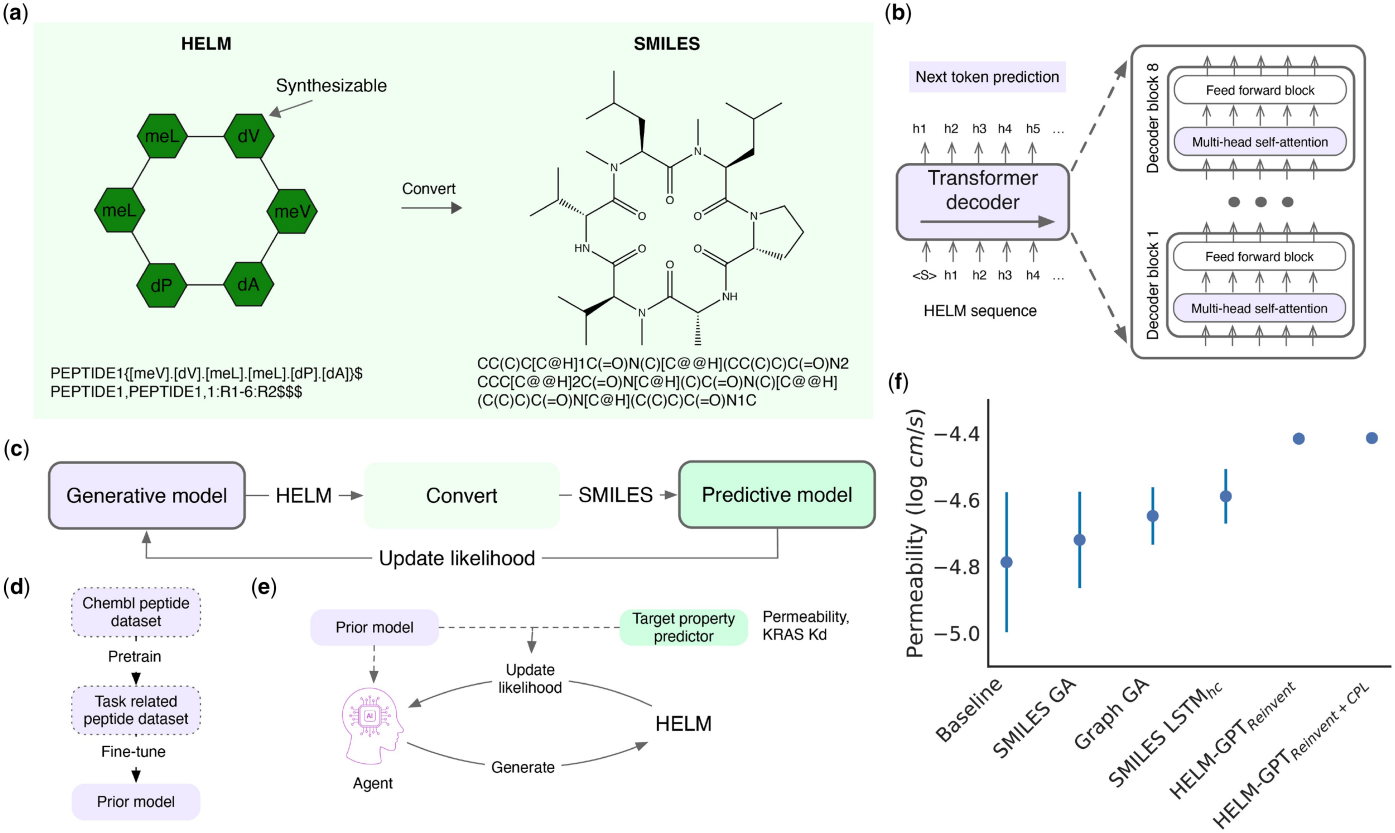
Overview of the HELM-GPT pipeline. (a) The HELM and the simplified molecular input line entry system (SMILES) representations of an example molecule, with the corresponding graph presentation presented. The monomers of the HELM can be predefined synthesizable non-natural amino acids. HELM is converted to SMILES for evaluation by predictive models, which requires SMILES as input to obtain the molecular features. (b) Illustration of the HELM-GPT architecture. It is a Transformer decoder with eight decoder blocks. The HELM sequences are tokenized to serve as input for the GPT, which is trained through the next token prediction. (c) Overview of the HELM-GPT workflow. A generative model was used to generate HELM sequences, which were then converted to SMILES to serve as input for predictive models. The scores of the predictive model serve as the feedback to update the generative model to increase the likelihood of generating the HELM sequences with desirable scores. (d) The workflow to pretrain and finetune the prior model, which is used to initiate the agent in RL. (e) Illustration of the RL process to train the agent model. During each RL step, the agent model is used to generate a batch of HELM sequences, which are then evaluated by the prior model and the property predictor to calculate the prior likelihoods and the property scores. The prior likelihood and the property scores are combined to update the likelihood of the agents to generate the HELM sequences. (f) The performance of the HELM-GPT model for macrocyclic peptide permeability optimization. HELM-GPT was able to achieve the best performance, though it is constrained into the HELM space with predefined monomers. The HELM-GPT model trained with added contrastive preference learning (CPL) loss has an enhanced target property optimization performance than the agent trained only with the Reinvent loss. GA, genetic algorithm; LSTM, long-short term memory.

In the realm of bio-therapeutics, a universal language is essential for capturing the intricate details of these complex molecules. The hierarchical editing language for macromolecules (HELM), originating from Pfizer in 2012 (Zhang *et al.* 2012), has become a widely adopted standard for representing various biotherapeutics, including peptides, RNA, RNA-peptide conjugates, antibody-drug conjugates, and even macrocyclic peptides (Li *et al.* 2023). HELM first defines the fundamental units–natural and non-natural amino acids–as monomers and then assembles them into sequences, known as simple polymers. Simple polymers can be linked through monomer bonds, forming intricate structures, called complex polymers. This allows HELM to represent a wide range of macromolecules with varying complexities. HELM offers a compact yet precise representation, as illustrated in Fig. 1a. This clarity makes it ideal for public databases, like ChEMBL (Mendez *et al.* 2019), and pharmaceutical companies around the globe.

This study presents the development of a method called HELM-GPT for designing macrocyclic peptides. The input sequence representation for this method is HELM, which is used with a generative pre-trained transformer (GPT) model (Radford *et al.* 2018), as shown in Fig. 1a and b. The GPT model is pre-trained on HELM sequences from the ChEMBL database (Mendez *et al.* 2019) to learn the HELM rules and then fine-tuned on high-scoring molecules for reinforcement learning (RL). To convert the HELM of a cyclic peptide into SMILES, which is the common input for the prediction of molecular properties, a HELM-to-SMILES conversion tool is developed, as shown in Fig. 1c. To finetune the HELM-GPT model and generate HELM sequences with desirable properties, a new contrastive preference learning (CPL) loss is proposed in combination with the Reinvent loss (Olivecrona *et al.* 2017, Hejna *et al.* 2024). One focus of the experiments is to design macrocyclic peptides targeting the intracellular protein KRAS. Predictive models are built to predict the cell permeability and KRAS binding affinity of the cyclic peptides, and these predictors guide the GPT model to generate HELM sequences with improved properties. The results demonstrate that the HELM-GPT model generates valid HELM sequences with high novelty. The HELM-GPT agent models in RL trained with both the CPL and the Reinvent loss display state-of-the-art performance in optimizing cell permeability and KRAS binding affinity. In order to resolve the challenge of co-optimizing binding affinity and cell permeability, a step-by-step optimization strategy is proposed, which demonstrated its effectiveness in generating molecules that meet both requirements. Given the wide use of HELM in representing macromolecules, the HELM-GPT method has the potential for widespread application in *de novo* macromolecule therapeutic discovery.

## 2 Results

### 2.1 Generating valid peptides using GPT

To generate macrocyclic peptides containing non-natural amino acids using GPT, it is crucial to establish a sequential representation for peptides. Upon reviewing the literature on macrocyclic peptides, we have identified five common sequence representations, namely HELM (Zhang *et al.* 2012), SMILES (Weininger 1988), amino acid sequence, and two other sequence representations (Rezai *et al.* 2006a,b). Although SMILES is extensively used for chemical compounds, it is not succinct in representing peptides. On the other hand, amino acid sequence and other sequential representations are concise but incapable of representing intricate peptides that involve non-natural amino acids and chemical bonds that cyclize linear peptides. HELM presents a balance between compact and comprehensive, as shown in Fig. 1a, and was selected as the appropriate peptide representation for this study.

To train a GPT model on HELM sequences, we gathered HELM sequences from three sources. We acquired 22 040 HELM sequences from the ChEMBL database (Mendez *et al.* 2019) and an additional 7451 sequences from the CycPeptMPDB database (Li *et al.* 2023). Furthermore, we selected 226 HELM sequences of KRAS-related peptides from patents to augment our dataset. These collected sequences were utilized to train our GPT prior models. A GPT model was pretrained on the ChEMBL dataset, and subsequently fine-tuned on either the CycPeptMPDB dataset or a combined dataset containing both KRAS and CycPeptMPDB sequences for downstream tasks.

We used Moses metrics (Polykovskiy *et al.* 2020) and the synthetic accessibility score (SAscore) (Blaschke *et al.* 2020) to assess the performance of GPT models. Five Moses metrics (Polykovskiy *et al.* 2020) were used, including validity, uniqueness, novelty, diversity, and similarity to a nearest neighbor (SNN). Details of the metrics are described in Supplementary Method Subsection S3.2. Validity measures the fraction of valid molecules among 1000 generated HELM sequences. Uniqueness measures the fraction of unique molecules among all the valid ones. Novelty measures the fraction of molecules that are not present in the training set among the unique generated molecules. Diversity is calculated as one minus the average Tanimoto similarity between any pair of generated molecules. SNN measures the similarity of the generated molecules to the training molecules. Since these metrics require SMILES as input, we devised functions to convert HELM sequences into SMILES using RDKit (Landrum *et al.* 2013). Our monomer library of 3104 monomers was constructed by consolidating the ones from ChEMBL, CycPeptMPDB, and KRAS HELMs. Subsequently, we devised an algorithm to generate the molecule corresponding to a HELM sequence using the peptide monomers. Finally, we used RDKit to obtain the canonical SMILES of the generated molecules. In total, 1000 molecules were sampled from the ChEMBL dataset as a baseline, while 1000 molecules were sampled from the GPT models in evaluation.

Table 1 displays the assessment results of the HELM-GPT prior models. The model pretrained on the ChEMBL dataset exhibits a validity rate of 70.8%, a uniqueness rate of 89.0%, and a high novelty score of 88.9%. Furthermore, it shows a similar diversity and SAscore to the baseline sequences. In addition, the model showcases a high similarity to the training dataset, with a SNN score of 0.750. This suggests that the GPT model effectively grasps the HELM rules, generates valid HELM sequences, and understands the property distributions of the training sequences in the HELM space. Fine-tuning the HELM-GPT model on task-specific datasets leads to further enhancements in validity (83.9% and 100.0%, respectively) and uniqueness (91.3% and 100.0%, respectively). These fine-tuned models then served as prior models for subsequent optimization tasks.

**Table 1.** Evaluation of HELM-GPT prior models.^a^

Model	Validity	Uniquenesss	Diversity	SNN	Novelty	SAscore
Baseline_*ChEMBL*_	**1.000**	**1.000**	**0.768**	**1.000**	0.000	0.551
Prior_*ChEMBL*_	0.708	0.890	0.740	0.750	**0.889**	**0.564**
Prior_*CycPeptMPDB*_	0.839	0.913	0.595	0.975	0.461	0.438
Prior${\;}_{\mathrm{KRAS}+\mathrm{CycPeptMPDB}}$	**1.000**	**1.000**	0.499	0.957	0.400	0.450

a The best scores are highlighted in bold. Baseline_*ChEMBL*_ is 1000 molecules randomly sampled from the ChEMBL dataset. Prior_*ChEMBL*_, Prior_*CycPeptMPDB*_, and Prior${\;}_{\mathrm{KRAS}+\mathrm{CycPeptMPDB}}$ are the HELM-GPT prior models pretrained on ChEMBL, fine-tuned on CycPeptMPDB, or fine-tuned on both KRAS and CycPeptMPDB datasets; 1000 molecules were sampled for evaluation. The HELM-GPT models can generate valid HELM sequences. SNN, similarity to a nearest neighbor, SAscore, synthetic accessibility score.

### 2.2 Cell permeability optimization

In this study, our objective is to develop cyclic cell-penetrating peptides. To assess the permeability of a peptide, we have constructed a regression model using the CycPeptMPDB dataset (Li *et al.* 2023). This dataset comprises 7451 cyclic peptides, each with a recorded membrane permeability value on a log scale. A permeability of >−6 is considered high, while values <−6 are deemed low. Out of the total peptides, 5113 have high permeability, while 2338 have low permeability. Both the HELM and SMILES representations are available for these peptides, but for the purpose of building predictive models, we have utilized the SMILES representation as the input.

**Figure 2.**
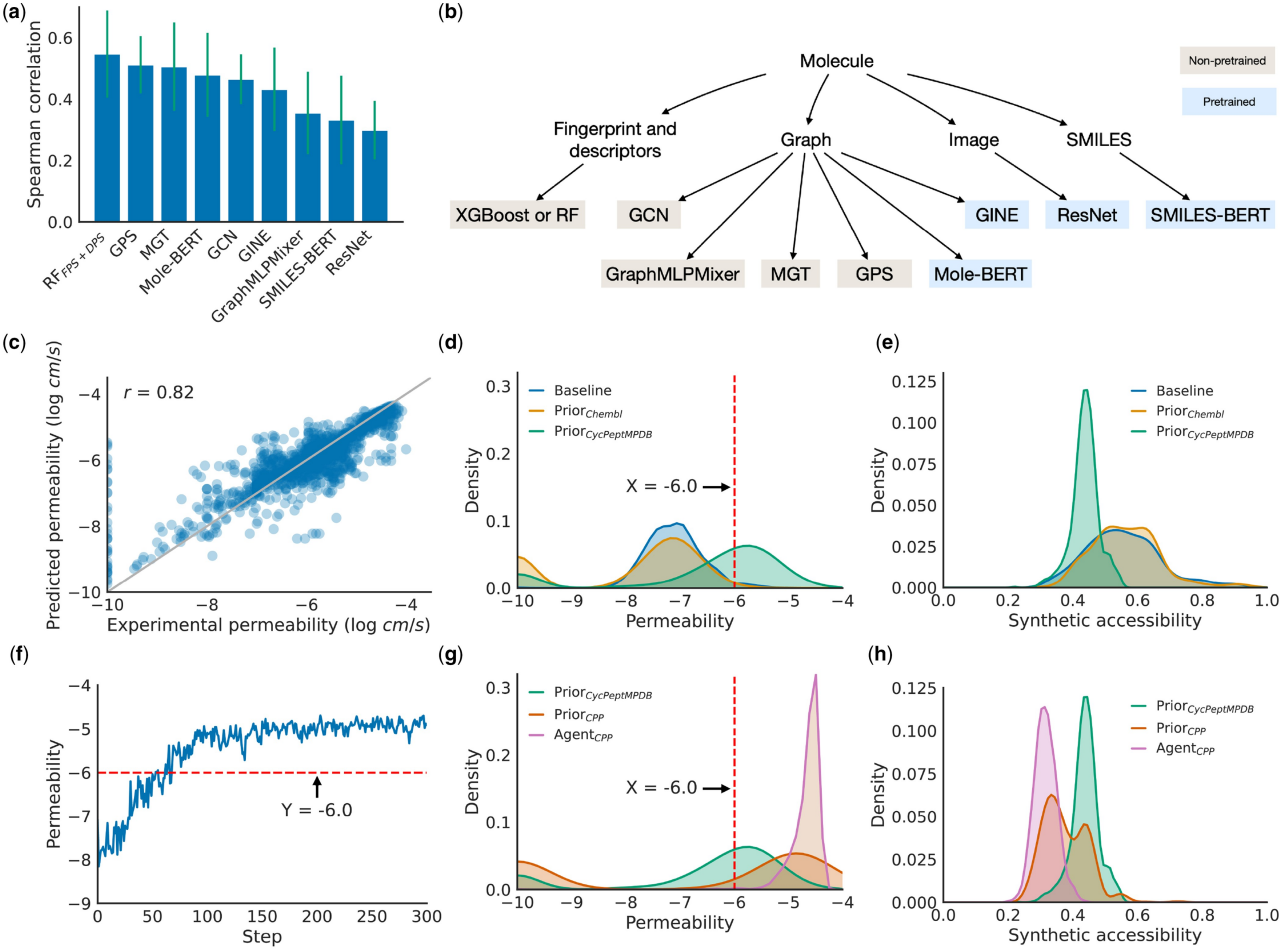
Optimizing cell permeability of macrocyclic peptides with HELM-GPT. (a) Comparison of predictive models for cell permeability prediction. Random forest model with fingerprints and descriptors as features showed a robust performance and was used in downstream tasks. (b) Classification of the predictive models evaluated. Fingerprints, descriptors, molecular graph, molecular image, and SMILES were evaluated as features. The state-of-the-art models were evaluated and compared. (c) Scatter plot of the predicted permeability and experimental permeability of macrocyclic peptides in the test dataset. The final predictive model achieved a Spearman correlation of 0.82. (d and e) The distribution of predicted permeability and synthetic accessibility of the molecules generated by the prior models. In total, 1000 molecules sampled from the ChEMBL database were evaluated as the baseline. (f) The mean predicted permeability of HELM-GPT generated molecules during the permeability optimization process. The model was able to generate molecules with high predicted permeability in 150 steps. (g and h) The distribution of predicted permeability and synthetic accessibility of the molecules generated by the final agent models in comparison to the prior models. The permeability curve was shifted towards higher permeability, while the synthetic accessibility was slightly decreased.

In order to build predictive models for molecules, we have explored various popular representations, including fingerprints and descriptors, molecular graphs, molecular images, and SMILES sequences. We evaluated state-of-the-art methods for each representation, as depicted in Fig. 2b. Among these methods, GINE, Mole-BERT, ResNet, and SMILES-BERT are pretrained methods, while GPS, MGT, and GraphMLPMixer are specifically designed for long-range graphs such as the molecular graphs of peptides. The results of these predictive models are displayed in Fig. 2a. Contrary to our initial expectations, graph-based methods did not demonstrate the highest performance on this task. Instead, a random forest model utilizing fingerprints and descriptors as features exhibited robust performance compared to the others. Long-range graph methods yielded competitive results, while SMILES and image-based methods under-performed in this context. Our analysis of the random forest model further revealed the significance of molecular descriptors, such as electrotopological state van der Waals surface area (EState_VSA) and the Wildman-Crippen logarithm of partition coefficient (MolLogP), as important features in the predictive model. Having conducted this analysis, we subsequently built a random forest model for permeability prediction. This model yielded a Spearman correlation coefficient of 0.82 on the test dataset. Figure 2c displays a scatter plot illustrating the relationship between the experimental permeability and the predicted permeability within the test dataset.

Then, we trained the HELM-GPT model specifically for generating cell-penetrating peptides. The GPT model was first fine-tuned on the CycPeptMPDB data and then further fine-tuned on the top 1000 molecules from the training datasets with the highest predicted permeability scores to serve as the prior model for the downstream RL process. The RL process was utilized to increase the likelihood of generating molecules with high permeability scores. The Reinvent loss (Olivecrona *et al.* 2017), a traditional loss used to update the agent in molecular optimization, was utilized in this process. We observed that this loss updates the likelihoods based on the predicted scores, without considering the pairwise preferences. So, we introduced a new loss term called contrastive preference learning (CPL) loss to include the pairwise preferences (Hejna *et al.* 2024). This loss would enable us to decrease the likelihood of molecules with undesirable scores by comparing the predicted scores of pairs of molecules and assigning binary preferences. The training curve of the agent model using the combined loss is depicted in Fig. 2f. Remarkably, within just 150 steps, the agent model successfully generated molecules with high predicted permeability. The permeability and synthetic accessibility distributions of the prior models and the agent model in the final step are illustrated in Fig. 2g and h, respectively. The agent model shifted the permeability distribution toward high permeability, while the synthetic accessibility slightly decreased. The effect of the CPL loss was also analyzed. Adding the CPL loss results a change in the log likelihoods which is positively related to the predicted permeability (*r* = 0.06).

To evaluate the models’ performance in permeability optimization, we used mean predicted permeability, SAscore, and three metrics from the Moses benchmark (novelty, diversity, and SNN) for comparison. We used 1000 random molecules from the ChEMBL dataset and 1000 molecules with the highest permeability from the ChEMBL and CycPeptMPDB dataset as baselines for comparison. The prior models and three other molecular optimization methods, SMILES genetic algorithms (GA) (Yoshikawa *et al.* 2018), Graph GA (Jensen 2019), and SMILES long short-term memory (LSTM) hill climbing (HC) (Neil *et al.* 2018), were assessed based on these metrics. SMILES GA evolves SMILES representations by introducing mutations following the SMILES context-free grammar. Graph GA evolves molecules at the graph level. SMILES LSTM HC uses LSTM as the policy network of the RL agent and optimizes the generation through iterative fine-tuning, using the best candidates from previous generations. The metrics were calculated based on the 1000 molecules with the highest permeability scores generated by these methods. It is worth noting that these three methods explore the SMILES or molecular graph space, and the molecules generated by them are not limited to peptides.

The comparison of these methods’ performance in permeability optimization is presented in Table 2. Despite being constrained by the HELM space, HELM-GPT with Reinvent and CPL loss (HELM-GPT${\;}_{\mathrm{Reinvent}+\mathrm{CPL}}$) achieved the highest performance in terms of permeability and novelty. SMILES GA, Graph GA, and SMILES LSTM HC showed superior performance in terms of diversity, which is understandable because they explore a wider chemical space compared to the HELM-GPT methods, which are constrained by predefined monomers. We expected the SAscores of the HELM-GPT-generated molecules to be higher since the monomers are predefined and synthesizable. However, we observed that SMILES GA, Graph GA, and SMILES LSTM HC achieved slightly better SAscores than the HELM-GPT methods. We suspect that the SAscore model, originally built for small molecules, may not be well suited to evaluate larger molecules such as cyclic peptides. In addition, SMILES GA, Graph GA, and SMILES LSTM HC tended to have better SNN than HELM-GPT models, which can be attributed to the greedy exploration process in these methods. The molecules with the highest permeability in the training dataset were used to generate the next generations in these methods, resulting in the final best molecules having high similarity to the original best candidates.

**Table 2.** Comparison of methods on macrocyclic peptide cell permeability optimization.^a^

Model	Predicted permeability	SAscore	Novelty	Diversity	SNN
Baseline_*ChEMBL*_	−7.104	0.551	0.000	**0.768**	**1.000**
Baseline_*Best*_	−**4.787**	0.375	0.000	0.654	**1.000**
Prior_*ChEMBL*_	−7.963	**0.564**	**0.889**	0.740	0.750
Prior_*CycPeptMPDB*_	−6.579	0.438	0.461	0.595	0.975
SMILES GA	−4.720	0.382	0.315	**0.667**	**0.922**
Graph GA	−4.648	**0.412**	0.573	0.659	0.818
SMILES LSTM_*HC*_	−4.589	0.336	0.588	0.593	0.809
HELM-GPT${\;}_{\mathrm{Reinvent}+\mathrm{CPL}}$	−**4.414**	0.314	**1.000**	0.368	0.772
HELM-GPT_*Reinvent*_	−4.416	0.314	0.999	0.379	0.774

a The best scores from the methods are highlighted in bold. The best scores from the baselines and prior models are also highlighted as a reference. SAscore, synthetic accessibility score; SNN, similarity to a nearest neighbor.

From this permeability optimization task, we observed that HELM-GPT successfully generated cyclic peptides with high novelty and predicted permeability scores. This capability would enable the model to explore the HELM space and design novel biotherapeutics for specific targets.

### 2.3 KRAS binding affinity optimization

**Figure 3.**
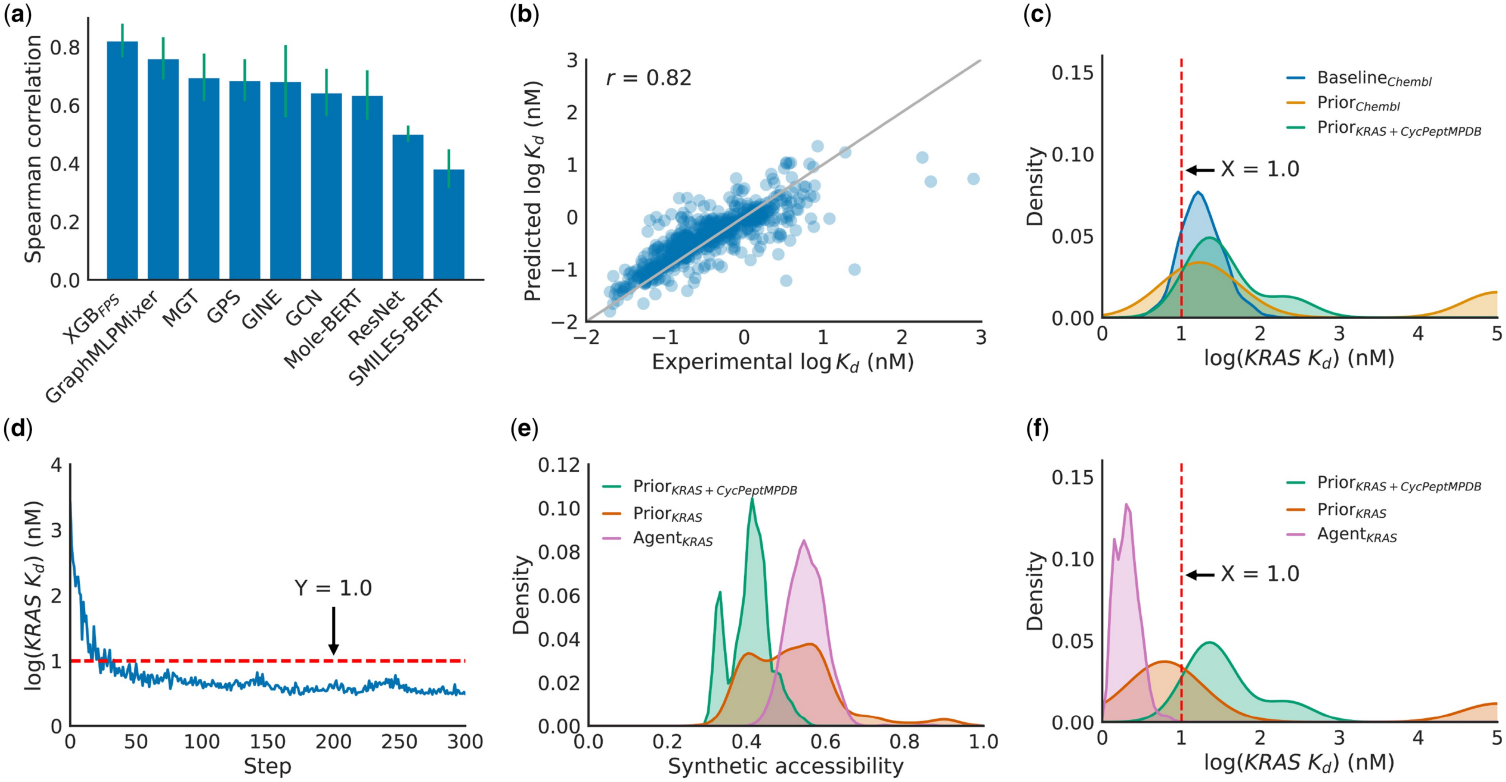
Optimizing KRAS binding affinity of peptides with HELM-GPT. (a) Comparison of predictive models for KRAS *K_d_* prediction. XGBoost model with fingerprints as features showed a robust performance and was used in downstream tasks. (b) Scatter plot of the predicted and experimental *K_d_* values of macrocyclic peptides in the test dataset. The model achieved a Spearman correlation of 0.82. (c) The distribution of predicted KRAS *K_d_* values of the molecules generated by the prior models. (d) The mean KRAS *K_d_* values of HELM-GPT generated molecules during the affinity optimization process. The model was able to generate molecules with low predicted KRAS *K_d_* within 100 steps. (e and f) The distribution of synthetic accessibility and KRAS *K_d_* values of the molecules generated by the final agent models in comparison to the prior models. The synthetic accessibility curve was shifted towards higher synthetic accessibility. The KRAS *K_d_* curve was shifted towards lower KRAS *K_d_* values, meaning an increase in KRAS binding affinity.

In the next task, our objective was to generate peptide binders to KRAS using HELM-GPT. However, before we could proceed with designing these peptides, we first needed to build a predictor that could assess the binding affinities of peptides against KRAS. To build this predictor, we collected data on the KRAS *K_d_* values of 2757 peptides from a patent (Kawada *et al.* 2023). The *K_d_* values were then converted into a logarithm scale for modeling. Once we had the data in place, we evaluated various predictive models using different input types such as fingerprints, descriptors, molecular graphs, molecular images, and SMILES sequences. The results of these evaluations are displayed in Fig. 3a. It was found that the XGBoost model using fingerprints as inputs achieved the best performance, although long-range graph models like GraphMLPMixer also performed well, albeit slightly inferior to XGBoost. We selected the XGBoost model as our predictor, and evaluated its performance on test datasets. The scatter plot of the model’s performance on the test datasets is shown in Fig. 3b. The final regression model achieved a strong Spearman correlation coefficient of 0.82.

To fine-tune the HELM-GPT model for generating peptides with high KRAS *K_d_* values, we used RL with the regression model serving as feedback. The HELM-GPT model was first fine-tuned on the KRAS and CycPeptMPDB dataset, and subsequently fine-tuned on a subset of 1000 molecules with the highest predicted *K_d_* values from the training dataset. During RL, similar to the permeability optimization task, we trained the agent models using both the Reinvent loss and the CPL loss. The learning curve of the agent model during the first 300 steps is depicted in Fig. 3d. Within 100 steps, the model was able to generate molecules with low KRAS *K_d_* scores. The distribution curves of synthetic accessibility and KRAS *K_d_* scores are shown in Fig. 3e and f, respectively. Both the synthetic accessibility and KRAS *K_d_* scores shifted into the desirable region, with an increase in synthetic accessibility and a decrease in KRAS *K_d_* scores.

To evaluate the performance of the HELM-GPT models, we compared the mean KRAS *K_d_* score, the mean SAscore, and three Moses metrics with other three methods, as done in the permeability optimization task. The baselines consisted of 1000 random peptides from the CheEMBL dataset and 1000 peptides with the best KRAS *K_d_* scores from the KRAS, ChEMBL, and CycPeptMPDB datasets. In addition, the top-performing molecules generated by the three molecular optimization methods (SMILES GA, Graph GA, and SMILES LSTM HC) were evaluated. For the HELM-GPT models, the metrics were calculated based on the evaluation of 1000 molecules with the highest KRAS *K_d_* scores explored by each agent model.

The results of the comparisons are presented in Table 3. All of the molecular optimization methods were successful in optimizing the KRAS *K_d_* scores, generating molecules with lower averaged KRAS *K_d_* scores than the best 1000 candidates in the training dataset. The HELM-GPT models achieved competitive performance in terms of KRAS *K_d_* scores, ranking second after the Graph GA method. In addition, HELM-GPT${\;}_{\mathrm{Reinvent}+\mathrm{CPL}}$ generally outperformed HELM-GPT_*Reinvent*_ on most metrics, except for diversity. This superiority of HELM-GPT${\;}_{\mathrm{Reinvent}+\mathrm{CPL}}$ suggests that the CPL loss is beneficial for training GPT models in the property optimization task. It is worth noting that SMILES GA and Graph GA achieved good performance on the binding optimization task. However, SMILES GA explores the SMILES space and Graph GA explores the molecular space, this will produce large portions of molecules that are not synthesizable based on current experimental technology. HELM-GPT is constrained to the HELM space with pre-defined synthesizable monomers, so the molecules generated will be synthesizable. This is an advantage of HELM-GPT in real-world peptide design tasks, where the synthetic accessibility of monomers is critical.

**Table 3.** Comparison of methods for KRAS-binding cyclic peptide generation.^a^

Model	Predicted KRAS *K_d_*	SAscore	Novelty	Diversity	SNN
Baseline_*ChEMBL*_	1.257	0.551	0.000	0.768	**1.000**
Baseline_*Best*_	**0.549**	0.552	0.000	**0.821**	**1.000**
Prior_*ChEMBL*_	2.316	**0.564**	**0.889**	0.740	0.750
Prior${\;}_{\mathrm{KRAS}+\mathrm{CycPeptMPDB}}$	1.553	0.450	0.400	0.499	0.957
SMILES GA	0.065	0.477	0.978	**0.851**	0.350
Graph GA	−**1.855**	0.464	**1.000**	0.485	0.163
SMILES LSTM_*HC*_	0.319	0.484	0.969	0.560	0.596
HELM-GPT${\;}_{\mathrm{Reinvent}+\mathrm{CPL}}$	0.038	**0.547**	**1.000**	0.301	**0.615**
HELM-GPT_*Reinvent*_	0.180	0.508	**1.000**	0.406	0.610

a The best scores from the methods are highlighted in bold. The KRAS *K_d_* score of HELM-GPT${\;}_{\mathrm{Reinvent}+\mathrm{CPL}}$ is underlined, which ranked the second. The best scores from the baselines and prior models are also highlighted as a reference. SAscore, synthetic accessibility score; SNN, similarity to a nearest neighbor.

### 2.4 Proposing novel peptides targeting intracellular KRAS

KRAS is a molecule located within the cell. To develop a macrocyclic peptide therapeutic targeting KRAS, the molecule needs to pass through the cell membrane and bind to KRAS with high affinity. Therefore, an ideal molecule should have both high permeability and a low KRAS *K_d_*. In previous tasks, we optimized permeability and KRAS *K_d_* individually using HELM-GPT. Here, we aim to utilize the capabilities of HELM-GPT to generate molecules with high permeability and low KRAS *K_d_* simultaneously. We set a permeability threshold of >−6.0 and a KRAS *K_d_* threshold of <10 (1.0 in logarithm scale).

**Figure 4.**
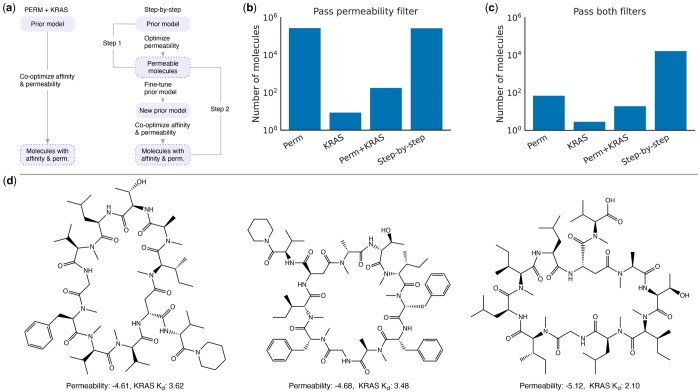
Proposing novel peptides targeting intracellular KRAS. Four strategies were explored to propose novel peptides targeting intracellular KRAS: optimizing permeability along (Perm), optimizing KRAS *K_d_* alone (KRAS), co-optimizing permeability and KRAS *K_d_* (Perm+KRAS), and step-by-step optimization by first optimizing permeability then optimizing the two property together (step-by-step). (a) Diagrams of the Perm+KRAS and the step-by-step optimization strategies. A new prior model was finetuned for co-optimization in the step-by-step strategy. (b) Number of the explored molecules passed the permeability filter. (c) Number of the explored molecules passed the permeability and KRAS *K_d_* filters. The threshold of permeability is >−6.0, and the threshold of KRAS *K_d_* is 10 (1.0 in logarithmic scale). (d) Examples of molecules generated by the step-by-step strategy that passed the permeability and the KRAS *K_d_* filters.

Intuitively, there are three strategies to generate molecules that meet these requirements using HELM-GPT. The first strategy is to optimize cell permeability, the second strategy is to optimize KRAS-binding affinity, and the third strategy is to optimize cell permeability and KRAS-binding affinity together. We can apply the property thresholds as filters to obtain the resulting molecules. However, our experiments showed that although HELM-GPT was able to optimize permeability and KRAS-binding affinity individually, it was challenging to optimize both properties simultaneously. Very few of the explored molecules during the optimization of one property were able to fulfill the requirements of the other property. For example, during the optimization of cell permeability, HELM-GPT explored 294 877 unique molecules, with 287 304 passing the permeability filter (Fig. 4b). However, only 75 molecules out of the explored ones passed the KRAS-binding affinity filter. Similarly, during the optimization of KRAS-binding affinity, only nine molecules passed the permeability filter. When the permeability and KRAS-binding affinity were co-optimized using HELM-GPT, as shown in the right diagram of Fig. 4a, 20 molecules were generated that passed both property filters, which was more than optimizing KRAS-binding affinity alone and, however, fewer than optimizing permeability alone. This is potentially due to the difficulty of improving permeability in the co-optimization process.

Given the difficulty observed in optimizing both properties simultaneously, we proposed a step-by-step optimization approach to generate molecules that pass both filters. We first optimized one property, in this case, permeability, and then performed the simultaneous optimization of both properties, as shown in the left diagram of Fig. 4a. The molecules that passed the filters during the permeability optimization were collected and used to fine-tune the prior model for downstream co-optimization. As a result, the HELM-GPT model generated 17 273 molecules that passed the two filters, which was a 785-fold improvement compared to directly running the co-optimization (Fig. 4c). Figure 4d shows three examples of molecules that passed the filters, along with their predicted permeability and KRAS *K_d_*. Although the predicted property scores cannot guarantee good experimental properties, generating a large set of molecules that passed the filters could be useful in designing libraries for identifying molecules with favorable experimental properties.

## 3 Discussion and conclusion

In this study, we developed HELM-GPT, a model for macrocyclic peptide design. Our results showed that HELM-GPT successfully learned the property distributions of the training dataset and generated valid HELM sequences. In addition, we demonstrated that the model can be trained using RL to optimize the properties of generated molecules. To address the molecular optimization problem, we introduced a CPL loss function, which outperformed the Reinvent approach in target property optimization. Despite being constrained by the HELM space of predefined monomers, HELM-GPT performed competitively with commonly used methods on SMILES and molecular graphs, including SMILES GA, Graph GA, and SMILES LSTM HC. Moreover, it was able to generate molecules with better predicted properties than the best molecules in the training datasets. Finally, we proposed a step-by-step strategy to co-optimize cell permeability and KRAS binding affinity. This strategy showed good results in the co-optimization task.

One crucial aspect of applying HELM for biomolecular design is defining the monomer space. For a design project, a set of monomers must be selected to suit the intended purpose. In our study, we combined monomers from the KRAS dataset, the ChEMBL, and CycPeptMPDB database to construct our monomer set. As chemical technologies continue to advance, it is necessary to expand the set of synthesizable monomers to explore additional possibilities.

In our experiments, we discovered that pretraining the prior model with molecules possessing desirable properties gave the agent model valuable prior knowledge to navigate the HELM space. In both the cell permeability and KRAS binding affinity optimization tasks, the models fine-tuned on molecules with the best properties yielded superior results compared to those without the fine-tuning. This improvement can be attributed to the learning of monomer preferences during the pretraining process. For tasks involving prior knowledge of monomer preferences, we believe that pretraining a HELM-GPT model on HELMs containing such monomers would facilitate knowledge transfer to the GPT model.

Another critical consideration when using the HELM-GPT approach is building reliable property predictors to guide property optimization. High-quality property predictors are essential for successful optimization tasks; otherwise, the HELM-GPT agent model may generate many false positives.

In the co-optimization of cell permeability and KRAS *K_d_*, we encountered difficulties in simultaneously optimizing the two properties using HELM-GPT. One potential reason is that the two properties are unrelated and possess distinct molecular patterns. To address this issue, we proposed a step-by-step strategy, gradually adding one property at a time. This step-by-step strategy showed good results in the co-optimization of cell permeability and KRAS *K_d_*. We believe this strategy can be applied to scenarios involving optimization of multiple properties beyond two.

Lastly, HELMs can be used to represent various types of macromolecules, including peptides, RNAs, RNA-peptide conjugates, and antibody-drug conjugates. The dataset for these macromolecule therapeutics is expanding (Mendez *et al.* 2019), and deep learning methods for predicting their properties are also evolving (Chen *et al.* 2021). With the accumulating data and the availability of high-quality property predictors, we believe that the HELM-GPT method has the potential for widespread application in macromolecule design.

## Supplementary Material

btae364_Supplementary_Data

## Data Availability

The HELM-GPT method and the associated data used in this article are available on GitHub, https://github.com/charlesxu90/helm-gpt.
