# Supplementary material for: HELM-GPT: de novo macrocyclic peptide design using generative pre-trained transformer
Source: Bioinformatics. 2024 Jun 12;40(6):btae364. doi: 10.1093/bioinformatics/btae364 (PMC11256930; doi:10.1093/bioinformatics/btae364)
Supplement: btae364_Supplementary_Data [file btae364_supplementary_data.pdf]

Supplementary information

HELM-GPT: de novo macrocyclic peptide design using generative pre-trained transformer

1 Supplementary tables

Supplementary Table 1: Comparison of common sequence representation of peptides. Hierarchical editing language for macromolecules (HELM) can represent macrocyclic peptides and is concise in presentation. SMILES, the simplified molecular input line entry system.

Representation	Example	Advantages	Disadvantages
Amino acid sequence	INLKKLAKLAKKIL	Only support natural AAs	Does not support non-natural amino acids and cyclic peptides
Rezai et al. [19]	Leu-Leu-d-Leu-d-Pro-Pro-Tyr(OME)	Support non-natural peptides	Does not support complex cyclic peptides
Rezai et al. [20]	cyclo[d-Leu-d-Leu-Leu-d-Leu-Pro-Tyr]	Support end2end cyclic peptides	Does not support complex peptides
SMILES [22]	C/C=C/C[C@@H](C)[C@@H](O)[C@H]1C(=O)N [C@@H](CC)C(=O)N(C)CC(=O)N(C)[C@@H](CC(C) C)C(=O)N[C@@H](C(C)C)C(=O)N(C)[C@@H](CC(C) C)C(=O)N[C@@H](C)C(=O)N[C@H](C)C(=O)N(C) [C@@H](CC(C)C)C(=O)N(C)[C@@H](CC(C)C)C(=O) N(C)[C@@H](C(C)C)C(=O)N1C	Support complex peptides	Not concise for peptides
HELM [26]	PEPTIDE1{[Abu].[Sar].[meL].V.[meL].A.[dA].[meL]. [meL].[meV].[Me_Bmt(E)]}$PEPTIDE1,PEPTIDE1, 1:R1-11:R2$$$	Support complex peptides and concise	

Supplementary Table 2: Performance of the predictive methods on cell permeability regression.

Model	MAE (↓)	MSE (↓)	Spearman (↑)	Pearson (↑)
$RF_{FPS+DPS}$	**0.609 ± 0.027**	**1.025 ± 0.227**	**0.547 ± 0.142**	**0.415 ± 0.141**
GPS	0.811 ± 0.126	1.245 ± 0.098	0.512 ± 0.093	0.408 ± 0.094
MGT	0.644 ± 0.024	1.133 ± 0.169	0.506 ± 0.144	0.399 ± 0.115
Mole-BERT	0.651 ± 0.073	1.107 ± 0.272	0.479 ± 0.137	0.364 ± 0.110
GCN	0.707 ± 0.114	1.168 ± 0.181	0.465 ± 0.081	0.349 ± 0.075
GINE	0.679 ± 0.050	1.178 ± 0.177	0.432 ± 0.136	0.320 ± 0.118
GraphMLPMixer	0.738 ± 0.079	1.340 ± 0.209	0.355 ± 0.134	0.300 ± 0.112
SMILES-BERT	5.367 ± 0.143	29.976 ± 1.630	0.332 ± 0.144	0.259 ± 0.121
ResNet	0.821 ± 0.098	1.352 ± 0.091	0.299 ± 0.095	0.251 ± 0.074

Supplementary Table 3: Performance of the predictive methods on KRAS K_d regression.

Model	MAE (↓)	MSE (↓)	Spearman (↑)	Pearson (↑)
XGB_{FPS}	**0.250 ± 0.034**	**0.135 ± 0.030**	**0.822 ± 0.058**	**0.815 ± 0.057**
GraphMLPMixer	0.303 ± 0.024	0.182 ± 0.015	0.761 ± 0.073	0.761 ± 0.057
MGT	0.379 ± 0.052	0.263 ± 0.063	0.696 ± 0.082	0.674 ± 0.093
GPS	0.369 ± 0.028	0.239 ± 0.029	0.686 ± 0.072	0.676 ± 0.070
GINE	0.399 ± 0.090	0.276 ± 0.104	0.683 ± 0.124	0.674 ± 0.137
GCN	0.465 ± 0.101	0.361 ± 0.109	0.644 ± 0.081	0.623 ± 0.087
Mole-BERT	0.388 ± 0.037	0.262 ± 0.051	0.635 ± 0.085	0.620 ± 0.105
ResNet	0.455 ± 0.060	0.331 ± 0.085	0.502 ± 0.028	0.505 ± 0.049
SMILES-BERT	0.596 ± 0.092	0.595 ± 0.167	0.382 ± 0.067	0.379 ± 0.076

2 Supplementary figures

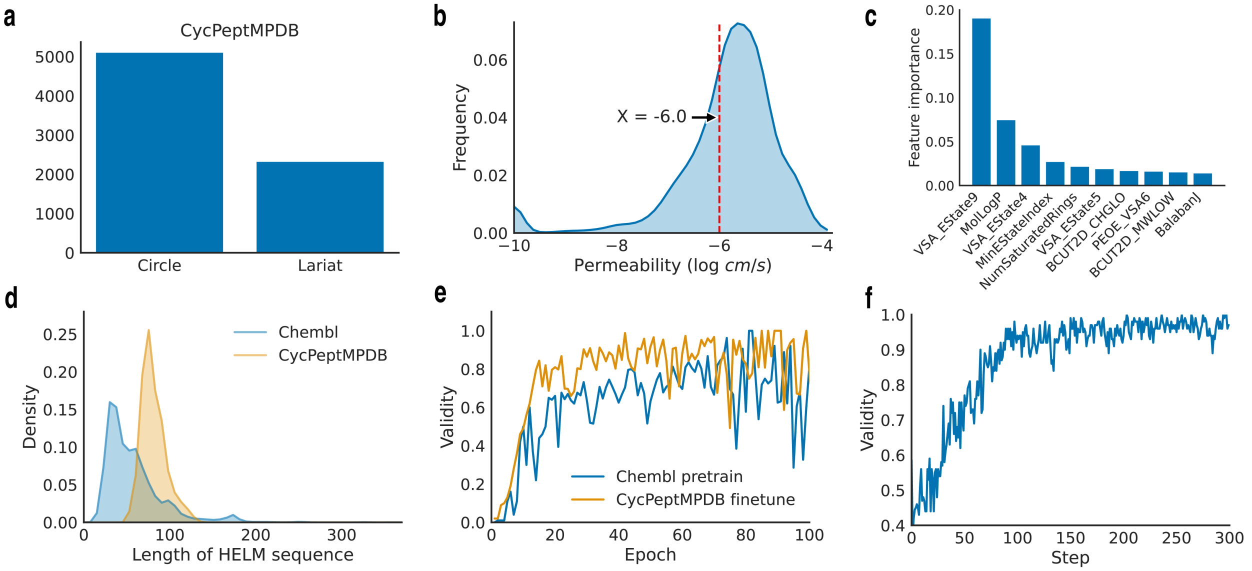

Supplementary Figure 1: Additional results of the cell permeability optimization task. a, The statistics of the CycPeptMPDB dataset. 5,115 cyclic and 2336 lariat peptides in the datasets. **b**, The permeability distribution of the peptides in the CycPeptMPDB dataset. The permeability value is in logarithm scale. -6.0 was selected as the threshold. **c**, The top ten most importance features in the random forest permeability regression model. All of them are molecular descriptors rather than fingerprints. **d**, The distribution of the lengths of HELM sequences from the ChEMBL and CycPeptMPDB dataset. **e**, The validity curve of the generated molecules during the pre-training and fine-tuning process of the prior model. 100 molecules were generated by each model for validity evaluation. **f**, The validity curve of the generated molecules during the reinforcement learning (RL) training process of the agent model. 100 molecules were generated in each RL step for evaluation.

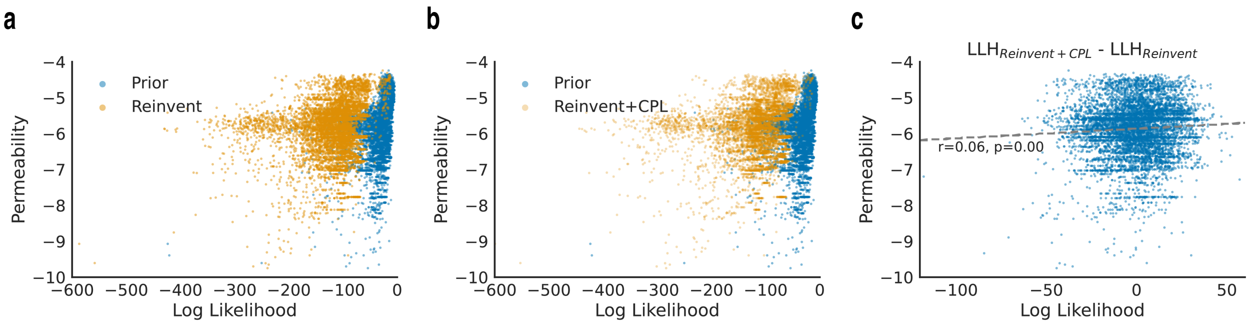

Supplementary Figure 2: The change of log likelihoods with and without the contrastive preference learning (CPL) loss. **a**, The scatter plot of prior and HELM-GPT$_{Reinvent}$ likelihoods of molecules in the CycPeptMPDB dataset. The predicted permeabilities were used as the Y axis to show the relationships. **b**, The scatter plot of prior and HELM-GPT$_{Reinvent+CPL}$ likelihoods of molecules in the CycPeptMPDB dataset. **c**, The scatter plot of likelihood differences of the two HELM-GPT models. This difference is contributed by the CPL loss and is positively related to the predicted permeability (r=0.06).

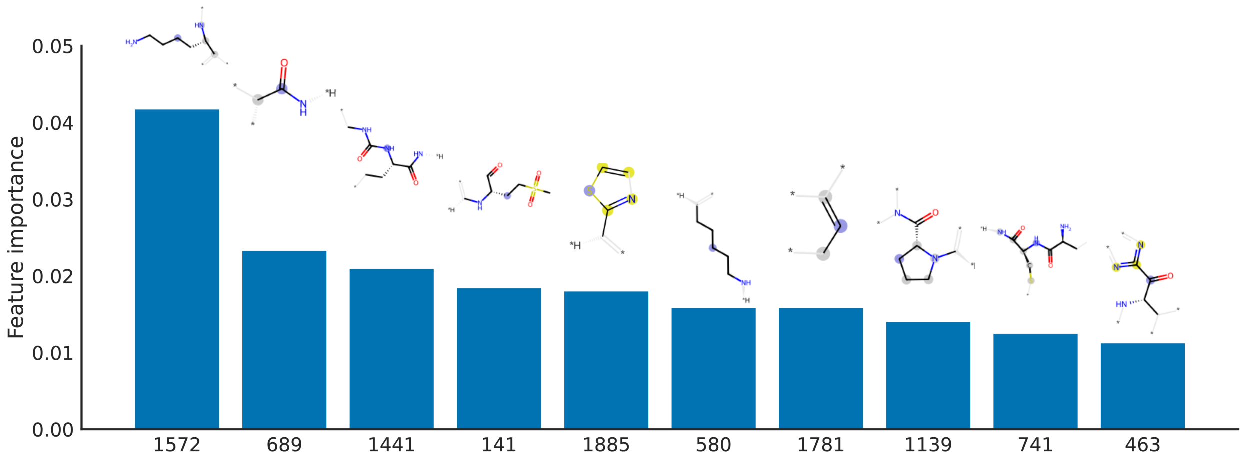

Supplementary Figure 3: The feature importance of the XGBoost KRAS K_d regression model. The top ten most important fingerprints are plotted.

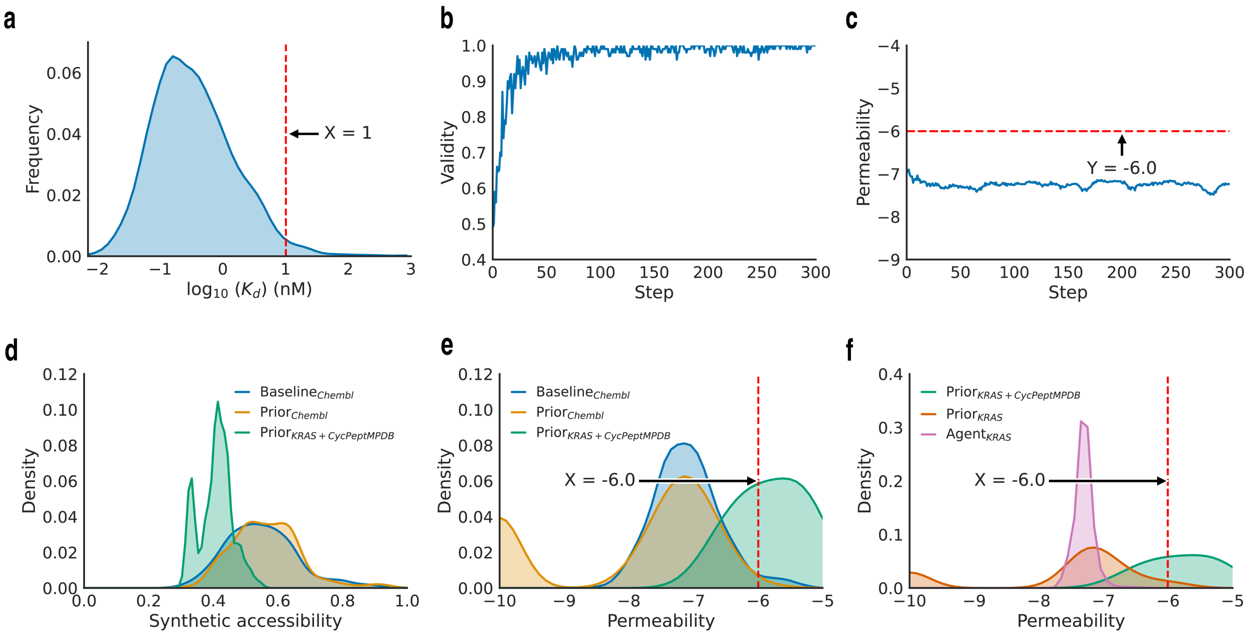

Supplementary Figure 4: Additional results of the KRAS K_d optimization task. **a**, The KRAS K_d distribution of the peptides in the KRAS dataset. The K_d value is in logarithm scale. 1.0 was selected as the threshold. **b**, The validity curve in the KRAS K_d optimization task. The agent model was able to generate molecules with high validity within 50 steps. **c**, The permeability curve in the KRAS K_d optimization task. The molecules generated during the KRAS K_d optimization process have poor permeability scores. **d**, The synthetic accessibility distribution of the molecules in the prior models. After fine-tuning, the synthetic accessibility was slightly decreased. **e**, The permeability distribution of the molecules in the prior models. **f**, The permeability distribution of the molecules generated by the agent models. After RL, the permeability is decreased comparing to the prior model.

3 Supplementary method

3.1 Data processing

In order to build the HELM-GPT models, we implemented a pretraining and fine-tuning strategy. The ChEMBL peptide dataset was used for pretraining the HELM-GPT model, while the CycPeptMPDB and KRAS datasets were used for fine-tuning. The ChEMBL dataset was obtained from the ChEMBL FTP site (release version 32). To obtain the peptides for pretraining, we followed the schema and selected molecules with the "protein" molecule type. Additionally, the ChEMBL monomers were downloaded from the same FTP site. We collected a total of 22,046 peptides with HELM sequences and 2,851 monomers. The CycPeptMPDB dataset, including 7,451 cyclic peptides and 312 monomers, was downloaded from the CycPeptMPDB website on March 27, 2023. Finally, the KRAS K_d dataset, consisting of 2,757 peptides with SMILES, was obtained from a patent [9]. To prepare the HELM model for fine-tuning, we manually extended the monomer library to convert 226 of the peptides into HELMs. After removing duplicated monomers, our final monomer library contained 3,104 monomers. Furthermore, we removed ChEMBL HELM sequences that were not covered by the final monomer library or were longer than 200, resulting in a dataset of 20,783 HELMs from ChEMBL.

3.2 Developing a tool to convert HELM into SMILES

To evaluate the validity of generated molecules and use the existing predictive methods, which require SMILES as inputs, we developed a tool to convert HELM into SMILES. The procedure of this tool is described in **Algorithm 1**. First, all smiles of the monomers were obtained from the monomer library, with associated R groups. Then, the bonds between the monomers were obtained, which are the connection relations between the R groups. The pseudo-code to obtain the the bonds of cyclic peptide is shown in **Algorithm 2**. In the linear peptide, the default connection between the R groups is from 'R2' to 'R1'. The connections of cyclic bonds are described in the HELM sequences. From these connections, we obtained the R groups occupied by the monomer bonds. After that, the unused R groups were restored to the original R groups of monomers. In the following steps, the monomers were connected together following the order in the linear peptide to obtain the SMILES of the linear peptide. Finally, the cyclic bonds were added to obtain the SMILES of the cyclic peptide. During these processes, RDKit [11] was used to connect two R groups following the monomer bonds or to restore the original R groups.

3.3 Training the HELM-GPT prior models

To train the HELM-GPT prior models, we first pretrain a GPT model using the ChEMBL dataset and then fine-tune the model using the CycPeptMPDB and KRAS datasets. For the pretraining process, the GPT model was trained on the ChEMBL dataset for 200 epochs. The maximum HELM length was set to 200, and the batch size was set to 1,024. During pretraining, we observed a decreasing loss in the first 34 epochs, followed by an increasing loss. Therefore, we selected the model checkpoint with the lowest loss, which was after the 34th epoch, for further analysis. We refer to this model as Prior_{ChEMBL}.

For the fine-tuning process, we trained the Prior_{ChEMBL} model on the CycPeptMPDB HELM dataset to create a prior model for cell permeability optimization. The model was trained for 200 epochs, and we again observed a decreasing-then-increasing trend in the loss. The model checkpoint after the 20th epoch, which had the lowest loss, was chosen for further analysis. This model is referred to as $\text{Prior}_{CycPeptMDB}$.

Similarly, to create a prior model for KRAS-binding peptide design, we fine-tuned the Prior_{ChEMBL} model on the CycPeptMPDB and KRAS datasets. The model checkpoint after the 7th epoch, which had the lowest loss, was selected for further analysis. This model is named $\text{Prior}_{KRAS+CycPeptMDB}$.

The prior models were initialized with eight layers, each with an embedding dimension of 256 and eight attention heads. AdamW optimizer with a learning rate of 0.001 was used to train the prior models. The training was conducted on a workstation with two A100 80G GPUs.

To evaluate the performance of the prior models, we calculated five Moses metrics [17], including validity, uniqueness, novelty, diversity, and similarity to a nearest neighbor (SNN). Validity measures the fraction of valid molecules among 1,000 generated HELM sequences. Uniqueness measures the fraction of unique molecules among all the valid ones. Novelty measures the fraction of molecules not present in the training

Algorithm 1 An algorithm to obtain peptide SMILES from HELM

```
Require:
  monomers2smi_dict                                    ▷ A dict of [monomer_name] - [SMILES] mapping
  monomers2r_groups_dict      ▷ A two-level dict of [monomer_name] - [R_group_id] - [R_group] mapping

  function GET_LINEAR_PEPTIDE(monomer_smis)                     ▷ Get SMILES of the linear peptide
      for idx, monomer in enumerate(monomer_smis) do
          if idx = 0 then
              smi ← monomer
          else
              smi ← COMBINE_FRAGMENTS(smi, monomer)             ▷ The function is adapted from RDKit
          end if
      end for
      Return smi
  end function

  function CYCLIZE_LINPEP_FROM_SMI(pep_smi, cyclic_link)           ▷ Add the cyclic_link in the SMILES
      start, end ← cyclic_link.split('-')
      pep_smi ← CONNECT_MAPPED_ATOMS(pep_smi, start, end)       ▷ The function is adapted from RDKit
      Return pep_smi
  end function

  function CYCLIZE_LINPEP_FROM_HELM(linear_helm, cyclic_linker)
              ▷ Get SMILES sequences based on the prepossessed linear_helm and cyclic_linker.
                    ▷ Example: linear_helm, {[dL].[dL].L.[dL].P.Y}; cyclic_linker, 1:R1-6:R2

                                                                   ▷ Get the list of monomers
      monomer_list ← linear_helm[1:-1].replace('[', '').replace(']', '').split('.')
      monomer_smis ← [deepcopy(monomers2smi_dict[monomer]) for monomer in monomer_list]

                                             ▷ Restore the unused R groups in monomer SMILES
      monomer_r_groups ← [deepcopy(monomers2r_groups_dict[monomer]) for monomer in monomer_list]
      monomer_links ← GET_LINKS_BETWEEN_MONOMERS(monomer_smis, cyclic_link)
      monomer_smis ← RESTORE_UNUSED_RGROUP(monomer_smis, monomer_r_groups, monomer_links)

      pep_smi ← GET_LINEAR_PEPTIDE(monomer_smis)                ▷ Merge the monomers one-by-one
      pep_smi ← CYCLIZE_LINPEP_FROM_SMI(pep_smi, cyclic_link)             ▷ Add the cyclic bonds
      Return pep_smi
  end function

  function GET_CYCPEP_SMI_FROM_HELM(helm)      ▷ helm: the HELM sequence of a peptide, example:
PEPTIDE1{[dL].[dL].L.[dL].P.Y}$PEPTIDE1, PEPTIDE1,1:R1-6:R2$$$
      helms ← helm.split('$')                                     ▷ Split HELM by the '$' symbol
      pep_idx ← helms[0].index('{')                    ▷ Get the start index of a peptide sequence
      linear_helm ← helms[0][pep_idx:]          ▷ Get the linear peptide sequence within the HELM
      cyclic_linker ← helms[1].split(',')[2] if ',' in helms[1] else None    ▷ Obtain the cyclic bond
      smi ← CYCLIZE_LINPEP_FROM_HELM(linear_helm, cyclic_linker)                ▷ Call the function
      Return smi
  end function
  smi ← GET_CYCPEP_SMI_FROM_HELM(helm)                             ▷ Example call of the function
```

set among the unique generated molecules. Diversity is calculated as one minus the average Tanimoto similarity between any pair of generated molecules. SNN measures the similarity of the generated molecules to the training molecules. It is calculated by finding the maximum Tanimoto similarity between each generated molecule and all molecules in the training dataset, and then averaging the maximum values. A higher SNN indicates greater similarity between the generated and training molecules. Additionally, synthetic accessibility score (SAscore) was calculated to measure the difficulty of synthesizing the generated molecules. It was obtained by using a predictive model [1] to predict the synthetic accessibility of each generated molecule. The mean synthetic accessibility score of the generated molecules was calculated as the SAscore. A higher SAscore indicates greater synthetic accessibility of the generated molecules.

Algorithm 2 Obtain all bonds between monomers in a HELM

```
function GET_LINKS_BETWEEN_MONOMERS(monomer_smis, cyclic_link)          ▷ Record the monomer bonds
    monomer_links = {}

    function ADD_LINK(source_idx, source_r_group, target_idx, target_r_group)    ▷ Add the monomer bonds
        if source_idx not in monomer_links then                          ▷ Add link to source monomer
            monomer_links[source_idx] ← {source_r_group : (target_idx, target_r_group)}
        else
            monomer_links[source_idx][source_r_group] ← (target_idx, target_r_group)
        end if
        if target_idx not in monomer_links then                          ▷ Add link to target monomer
            monomer_links[target_idx] ← {target_r_group : None}
        else
            monomer_links[target_idx][target_r_group] ← None
        end if
    end function

    for idx in range(len(monomer_smis) − 1) do
        ADD_LINK(idx, '_R2', idx + 1, '_R1')
    end for

    function GET_IDX_RGROUP(end)          ▷ An end contains the index and the R group of the monomer
        idx, r_group ← end.split(':')
    end function

    if cyclic_link then                                                  ▷ Add cyclic link
        source, target = cyclic_link.split('-')
        source_idx, source_r_group = GET_IDX_RGROUP(source)
        target_idx, target_r_group = GET_IDX_RGROUP(target)
        ADD_LINK(source_idx, source_r_group, target_idx, target_r_group)
    end if

    Return monomer_links
end function
```

3.4 Building predictive models for peptide permeability and KRAS binding affinity

To optimize peptides for permeability and KRAS binding affinity, we developed predictive models to predict these two properties. We employed various models, including ones using fingerprints, descriptors, molecular graphs, molecular images, or SMILES as inputs. The RDKit package was used to generate fingerprints, descriptors, and molecular images [11]. We utilized XGBoost [3] and random forest [2] regressors with fingerprints, descriptors, or a combination of both as inputs to compare their performance. Random forest was implemented using Scikit-learn [16], and XGBoost was implemented using the XGBoost package [3]. Among the two models, we selected the one with superior performance for final evaluation. For evaluating graph-based methods in predicting these two properties, we considered popular methods such as GCN [10] and GINE [7] as well as long-range graph methods including GPS [18], MGT [14], and GraphMLPMixer [4], and pretrained Mole-BERT [23]. GCN and GINE were implemented based on the original work by GINE [7], while GPS, MGT, Mole-BERT, and GraphMLPMixer were adapted from their respective original repositories. Additionally, we implemented image-based and SMILES-based methods following the approaches of ImageMol [25] and SMILES-BERT [21], respectively.

For the permeability prediction task, we used the CycPeptMPDB dataset [12] containing a total of 7,451 peptides to train our model for cell-permeability regression. In the KRAS binding affinity prediction task, we utilized 2,757 peptides from a patent [9] to train the model for KRAS Kd regression. To ensure robustness, all models underwent cross-validation with five different scaffold splits. Finally, we calculated the mean and standard deviation of the Spearman correlation on the test dataset for comparison purposes.

3.5 Peptide property optimization

3.5.1 RL formulation of HELM sequence optimization

The HELM-GPT model was employed to enhance the properties of peptide permeability and KRAS binding affinity. This was accomplished by training the GPT model through reinforcement learning (RL). In the RL formulation, the state is the current HELM sequence generated, the action is to sample the next token. The task was episodic and ended when an end token was sampled or the maximum length was reached. The reward was calculated after the full sequence was sampled and scored by the predictive models.

Fig. 1e illustrates the RL process, in which the agent model was updated to increase the likelihood of generating HELM sequences with improved properties. The Reinvent method [15] initially proposed for small molecule generation was adapted for HELM sequence generation, with GPT serving as the policy network. The prior log-likelihood $L(x)_{prior}$ is combined with the predicted score $S(x)$ to update the agent's likelihood $L(x)_{agent}$, as shown in Equation 1.

$$\mathcal{L}_{Reinvent} = [L(x)_{prior} + \sigma S(x) - L(x)_{agent}]^2 \tag{1}$$

Upon closer examination of the Reinvent loss, we found it to favor the global trend of the predicted scores, but the local pairwise differences was not well captured. So, we considered the potential to update the likelihoods based on pairwise preferences to learn local differences in the scores.

A recent study introduced a new approach called contrastive preference learning (CPL) for updating the policy model [5]. CPL involves calculating a regret between a pair of samples and mapping this regret to a binary preference. Motivated by this learning paradigm, we defined a contrastive preference loss for our scenario. The regret was determined by the difference between the prior likelihood and the agent likelihood for a given sample, as shown in Equation 2.

$$Regret(x) = L(x)_{prior} - L(x)_{agent} \tag{2}$$

The preference between a pair of samples (x_1, x_2) was determined by comparing their scores, with a value of one indicating a preference to the first sample and zero indicating preference for the second sample.

$$Pref(x_1, x_2) = Boolean(S(x_1) > S(x_2)) \tag{3}$$

The CPL loss was defined as a biased binary cross-entropy loss [5], allowing for the learning of local preferences between pairs of samples and updating the likelihoods accordingly.

$$\mathcal{L}_{CPL} = Biased\_bce\_with\_logits(Regret(x_1), Regret(x_2), Pref(x_1, x_2)) \tag{4}$$

Finally, our loss function was defined as the sum of the Reinvent loss and the CPL loss, incorporating both global scores and local pairwise preferences as feedback for updating the agent.

$$\mathcal{L} = \mathcal{L}_{Reinvent} + \mathcal{L}_{CPL} \tag{5}$$

3.5.2 Baseline methods for comparison

In order to evaluate the performance of HELM-GPT on molecular optimization tasks, we established two baselines and selected three other molecular optimization methods for comparison. The first baseline, referred to as Baseline$_{ChEMBL}$, consisted of 1,000 randomly sampled HELMs from the ChEMBL dataset. The second baseline, Baseline$_{Best}$, included 1,000 HELMs with the best target properties from the ChEMBL, CycPeptMPDB, and KRAS datasets.

The three molecular optimization methods chosen for comparison were SMILES genetic algorithms (GA) [24], Graph GA [8], and SMILES LSTM hill climbing (HC) [6, 13]. SMILES GA evolved SMILES sequences of top-performing molecules through a genetic algorithm, incorporating mutations according to the SMILES context-free grammar. It was run with a population size of 1,000, a gene size of 300, and for 500 generations to identify the best 1,000 molecules. Graph GA evolved molecules at the graph level, applying crossover on the best-performing molecules from the previous generations and randomly mutating the new molecules. A pool size of 1,000 was used to identify the best 1,000 molecules within 500 generations. SMILES LSTM

HC used LSTM to generate new SMILES sequences and iteratively fine-tuned the LSTM model with the best candidates from the previous generation. 1,024 molecules were generated and the top-scoring 1,000 molecules were selected to fine-tune the model in each step. It was trained for 20 steps to obtain the 1,000 best performing molecules. Each method operated within either the SMILES or molecular graph space and had the ability to generate valid molecules, but their synthetic accessibility in real-world applications was not guaranteed.

3.5.3 Permeability optimization task

To optimize cell permeability, we used a random forest model as the property predictor. This model utilized fingerprints and descriptors as features and demonstrated superior performance in permeability prediction. The predicted permeability scores were used directly in the three baseline methods. However, in the HELM-GPT method, we transformed these scores into the range of zero to one using a sigmoid function (Equation 6), where the low and high scores were set as -8.0 and -4.0, respectively.

$$Sigmoid(x, l, h) = \frac{1}{1 + 10^{-k * \frac{x - \frac{h+l}{2}}{h-l}}} \tag{6}$$

To improve optimization speed, the GPT prior model was fine-tuned using the top-scoring HELMs from the training dataset before the reinforcement learning (RL) process. The agent model was then trained for 3,000 RL steps, with 100 molecules generated during each step. The top 1,000 molecules generated during the RL process were used for evaluation.

3.5.4 KRAS binding affinity optimization task

For the optimization of KRAS binding affinity, a XGBoost model was used as the property predictor. This model employed fingerprints as features and achieved the best performance in KRAS K_d prediction. Since a lower score is desired for KRAS K_d, a reverse sigmoid transformation (Equation 7) was applied to the predicted score to obtain a transformed score, where higher scores were indicative of better binding affinity. The reverse sigmoid function not only reversed the score, but also ensured it fell within the range of zero to one, which is desirable in the HELM-GPT optimization process.

$$Reverse\_sigmoid(x, l, h) = \frac{1}{1 + 10^{k * \frac{x - \frac{h+l}{2}}{h-l}}} \tag{7}$$

Similar to the permeability task, the GPT prior model was fine-tuned on the top-scoring HELMs from the training dataset before the RL process. The agent model was trained for 3,000 steps, with a batch size of 100. The top 1,000 molecules generated during the RL steps were used for evaluation.

3.6 Generating peptides with both good permeability and KRAS binding affinity

The final task involved generating molecules with both high permeability and low KRAS K_d using HELM-GPT. To accomplish this, four strategies were explored: optimizing permeability alone, optimizing KRAS K_d alone, co-optimizing KRAS K_d and permeability, and step-by-step optimization. Single property optimization was performed as described in the previous Subsections, with molecules passing the filters considered successful.

For co-optimization of KRAS K_d and permeability, the transformed scores of the two properties were added together to form a scoring function. The prior model for this step was initialized with the prior model fine-tuned on top scoring molecules from the KRAS and CycPeptMPDB datasets.

In the step-by-step optimization strategy, the first step involved optimizing permeability, which was the same as the permeability optimization task. The second step involved co-optimization of both properties, employing the same configuration as the co-optimization strategy described earlier, with the exception of the prior model. After fine-tuning on KRAS and CycPeptMPDB datasets, the prior model was further fine-tuned on the successful molecules generated during the permeability optimization step.

References

[1] T. Blaschke, J. Arús-Pous, H. Chen, C. Margreitter, C. Tyrchan, O. Engkvist, K. Papadopoulos, and A. Patronov. Reinvent 2.0: an ai tool for de novo drug design. *Journal of chemical information and modeling*, 60(12):5918–5922, 2020.

[2] L. Breiman. Random forests. *Machine learning*, 45:5–32, 2001.

[3] T. Chen and C. Guestrin. Xgboost: A scalable tree boosting system. In *Proceedings of the 22nd acm sigkdd international conference on knowledge discovery and data mining*, pages 785–794, 2016.

[4] X. He, B. Hooi, T. Laurent, A. Perold, Y. LeCun, and X. Bresson. A generalization of vit/mlp-mixer to graphs. In *International Conference on Machine Learning*, pages 12724–12745. PMLR, 2023.

[5] J. Hejna, R. Rafailov, H. Sikchi, C. Finn, S. Niekum, W. B. Knox, and D. Sadigh. Contrastive prefence learning: Learning from human feedback without rl. *arXiv preprint arXiv:2310.13639*, 2023.

[6] S. Hochreiter and J. Schmidhuber. Long short-term memory. *Neural computation*, 9(8):1735–1780, 1997.

[7] W. Hu, B. Liu, J. Gomes, M. Zitnik, P. Liang, V. Pande, and J. Leskovec. Strategies for pre-training graph neural networks. *arXiv preprint arXiv:1905.12265*, 2019.

[8] J. H. Jensen. A graph-based genetic algorithm and generative model/monte carlo tree search for the exploration of chemical space. *Chemical science*, 10(12):3567–3572, 2019.

[9] H. Kawada, K. Takano, T. Kotake, M. Kage, S. Hashimoto, M. Tamiya, Y. Wakamiya, R. Hayashi, and Y. Morita. A cyclic compound with selective inhibitory action on KRASOVER HRAS and NRAS, Dec. 2023.

[10] T. N. Kipf and M. Welling. Semi-supervised classification with graph convolutional networks. *arXiv preprint arXiv:1609.02907*, 2016.

[11] G. Landrum et al. Rdkit: A software suite for cheminformatics, computational chemistry, and predictive modeling. *Greg Landrum*, 8:31, 2013.

[12] J. Li, K. Yanagisawa, M. Sugita, T. Fujie, M. Ohue, and Y. Akiyama. Cycpeptmpdb: A comprehensive database of membrane permeability of cyclic peptides. *Journal of Chemical Information and Modeling*, 63(7):2240–2250, 2023.

[13] D. Neil, M. Segler, L. Guasch, M. Ahmed, D. Plumbley, M. Sellwood, and N. Brown. Exploring deep recurrent models with reinforcement learning for molecule design. 2018.

[14] N. K. Ngo, T. S. Hy, and R. Kondor. Multiresolution graph transformers and wavelet positional encoding for learning long-range and hierarchical structures. *The Journal of Chemical Physics*, 159(3), 2023.

[15] M. Olivecrona, T. Blaschke, O. Engkvist, and H. Chen. Molecular de-novo design through deep reinforcement learning. *Journal of cheminformatics*, 9(1):1–14, 2017.

[16] F. Pedregosa, G. Varoquaux, A. Gramfort, V. Michel, B. Thirion, O. Grisel, M. Blondel, P. Prettenhofer, R. Weiss, V. Dubourg, et al. Scikit-learn: Machine learning in python. *the Journal of machine Learning research*, 12:2825–2830, 2011.

[17] D. Polykovskiy, A. Zhebrak, B. Sanchez-Lengeling, S. Golovanov, O. Tatanov, S. Belyaev, R. Kurbanov, A. Artamonov, V. Aladinskiy, M. Veselov, et al. Molecular sets (moses): a benchmarking platform for molecular generation models. *Frontiers in pharmacology*, 11:565644, 2020.

[18] L. Rampášek, M. Galkin, V. P. Dwivedi, A. T. Luu, G. Wolf, and D. Beaini. Recipe for a general, powerful, scalable graph transformer. *Advances in Neural Information Processing Systems*, 35:14501–14515, 2022.

[19] T. Rezai, J. E. Bock, M. V. Zhou, C. Kalyanaraman, R. S. Lokey, and M. P. Jacobson. Conformational flexibility, internal hydrogen bonding, and passive membrane permeability: successful in silico prediction of the relative permeabilities of cyclic peptides. *Journal of the American Chemical Society*, 128(43):14073–14080, 2006.

[20] T. Rezai, B. Yu, G. L. Millhauser, M. P. Jacobson, and R. S. Lokey. Testing the conformational hypothesis of passive membrane permeability using synthetic cyclic peptide diastereomers. *Journal of the American Chemical Society*, 128(8):2510–2511, 2006.

[21] S. Wang, Y. Guo, Y. Wang, H. Sun, and J. Huang. Smiles-bert: large scale unsupervised pre-training for molecular property prediction. In *Proceedings of the 10th ACM international conference on bioinformatics, computational biology and health informatics*, pages 429–436, 2019.

[22] D. Weininger. Smiles, a chemical language and information system. 1. introduction to methodology and encoding rules. *Journal of chemical information and computer sciences*, 28(1):31–36, 1988.

[23] J. Xia, C. Zhao, B. Hu, Z. Gao, C. Tan, Y. Liu, S. Li, and S. Z. Li. Mole-bert: Rethinking pre-training graph neural networks for molecules. In *The Eleventh International Conference on Learning Representations*, 2022.

[24] N. Yoshikawa, K. Terayama, M. Sumita, T. Homma, K. Oono, and K. Tsuda. Population-based de novo molecule generation, using grammatical evolution. *Chemistry Letters*, 47(11):1431–1434, 2018.

[25] X. Zeng, H. Xiang, L. Yu, J. Wang, K. Li, R. Nussinov, and F. Cheng. Accurate prediction of molecular properties and drug targets using a self-supervised image representation learning framework. *Nature Machine Intelligence*, 4(11):1004–1016, 2022.

[26] T. Zhang, H. Li, H. Xi, R. V. Stanton, and S. H. Rotstein. Helm: a hierarchical notation language for complex biomolecule structure representation, 2012.
